# Expressed emotion in the South Asian diaspora living in the UK: A qualitative study

**DOI:** 10.1371/journal.pone.0280103

**Published:** 2023-11-27

**Authors:** Hira Salman Sharif, Syed K. Miah, Amrita Ramanathan, Naomi Glover, Madiha Shaikh

**Affiliations:** 1 Department of Psychiatry, University College London, London, United Kingdom; 2 Research Department of Clinical, Educational and Health Psychology, University College London, London, United Kingdom; 3 North East London NHS Foundation Trust, London, United Kingdom; Jawaharlal Institute of Postgraduate Medical Education and Research, INDIA

## Abstract

**Background:**

‘Expressed Emotion (EE)’ captures ways in which emotions are expressed within a family environment. Research has found that EE in families has an impact on psychiatric illness, in particular psychosis, such that it increases risk of relapse. EE was conceptualised by research conducted in the UK. Thus, behaviours defined as pathological were largely based on white samples adhering to UK norms. Cross-cultural variations have been found in EE and its relationship with clinical outcomes. A more culturally appropriate understanding of norms surrounding the EE across cultures is required.

**Aims:**

This study aims to use a bottom-up approach to provide a culturally specific understanding of family relationships and EE across ‘non-clinical’ UK-based South Asian families.

**Methods:**

Semi-structured interviews were conducted with 18 South Asian participants to explore their relationships with a significant other. Interviews were analysed using thematic analysis.

**Results:**

Four main themes were generated: expression of love, setting boundaries, inter-generational differences and acceptance.

**Conclusion:**

The findings indicate considerable cultural variability within EE and highlight the need to interpret EE in the context of socio-cultural norms. Whilst certain domains of EE that are considered pathological in Western contexts are present in the UK-based South Asian diaspora, these are perceived as less problematic, indicative of varying cultural norms.

## Introduction

Expressed Emotion (EE) is used to assess the overall quality of the family environment with a focus on social interactions in caregiving relationships [1]. Brown [2] characterised five components of EE: critical comments, hostility, emotional over-involvement (EOI), positive remarks and warmth. High EE is defined by increased EOI, hostility and criticism. Research has indicated that higher EE in families impacts patient outcomes across a range of mental health problems [3, 4]. In particular, high EE leads to poorer patient mental health outcomes and increases the risk of relapse in psychosis [5].

EE was conceptualised by research conducted in the UK by George Brown, Michael Rutter and colleagues in the late 1950s and 1960s whilst originally trying to determine whether emotions in regular family relationships could be accurately and objectively measured [6, 7]. Thus, behaviours defined as pathological were largely based on white samples adhering to UK norms. Cross-cultural variations have been found in EE and its relationship with clinical outcomes. Anglo-Americans, for example, display significantly higher EE, including more critical comments and hostility, as compared to their Mexican-American counterparts [8]. Moreover, a meta-analysis found that negative outcomes attributed to greater EOI were not observed cross-culturally [9]. Rosenfarb et al [10] found that in Black families, higher levels of criticism and intrusiveness were correlated with lower relapse rates. In these families, criticism was seemingly indicative of care and support which consequently decreased patient stress. Another study found UK caregivers of people with schizophrenia and dementia reported greater levels of high EE compared to Japanese caregivers of people with the same conditions [11]. Moreover, results showed stark differences in the median number of critical comments across ethnic groups (UK = 6.5 and Japanese = 2). Domain level differences across geographical regions have been highlighted by studies that showed Indian and Chinese samples having higher criticism and warmth compared to Danish and British samples [12, 13]. This may be because, unlike in Western cultures, in Chinese cultures, criticism is viewed as a symbol of concern [12].

A recent meta-analysis explored the distribution of EE and its domains across cultures, whilst also assessing the relationship between EE and psychotic relapse [14]. Results showed that exposure to high familial EE increased the chance of a relapse by 95% compared to low EE and suggests this relationship is universal. However, there were no significant differences in overall EE scores or domain level scores based on geographical regions. The authors note the categorisation of high and low EE may neglect normative family values and the complexity of culturally defining EE domains. In addition, multiple adjustments to scoring the CFI were made, based on cultural norms, especially for criticism, EOI and warmth. Thus, authors argue against a universal normative EE profile due to the presence of cultural variation in the scoring and interpretation of EE [14].

These findings challenge the universal relationship between high EE and risk of relapse, and thus interventions that aim to reduce EE in families may not always be necessary nor effective across cultures. As such, high levels of certain EE domains may not be detrimental in all cultural contexts. NICE guidelines have recommended using Family Interventions (FI) for psychosis aimed at reducing EE within families, and therefore, decreasing patients’ risk of relapse [15]. In the UK, FI is widely used with culturally-diverse populations. Since research has determined that EE varies significantly by culture, both in relation to patient outcomes and prevalence, FI aiming to reduce EE could have adverse effects in non-‘individualistic’ populations [16]. This approach may pathologise customary and even protective behaviours in certain cultures [17]. A greater understanding of EE and its domains across cultures is therefore necessary to ensure the application of culturally appropriate interventions.

In addition, studies have also found differences in EE rates within the same ethnic groups. For example, an early study comparing high EE rates in families caring for someone with psychosis, found significantly lower rates of high EE in India compared to UK and Denmark [13]. Moreover, when the Indian sample was separated into rural and urban inhabitants, high EE rates were considerably lower in rural families (8%) compared to urban (30%). Similar findings were highlighted by another study exploring EE in Chengdhu, China [12], which also noted that people living in the city displayed greater EE than people living in rural areas. Few studies have investigated EE in South Asian populations living in the UK. A study conducted by Hashemi and Cochrane [18] found higher levels of EE in families of British Pakistani [Muslim] patients compared to British Sikh and White families. In both Asian samples, EE did not predict relapse in individuals with psychosis. In a ‘non-clinical’ population (i.e. those without any psychiatric illness), higher levels of EE, especially EOI, were typical in Pakistani Muslims, a characteristic which differed even to Indian Sikhs. Criticism in these families was also significantly higher compared to White families, despite there being no significant differences in criticism in the clinical population. These results show that cultures that appear very outwardly similar may differ in their response to mental illness. They suggest that Pakistani families may generally have intense emotional interactions regardless of whether they are facing obvious stressors; aspects like higher EOI in these families may be indicative of cultural rather than pathological traits. Crucially, the study sample consisted of only Pakistani Muslim and Indian Sikh families; therefore, its application to other South Asian cultures is limited. Moreover, since existing conceptualisations of EE domains (criticism, hostility, EOI) were applied to the South Asian context, it limits the cultural underpinnings of factors that contribute to the quality of a family environment. A more culturally appropriate understanding of norms surrounding EE across cultures is required. This study aims to use a bottom-up approach to provide a culturally specific understanding of family relationships and EE across ‘non-clinical’ UK-based South Asian families.

## Methods

This study received ethical approval by the Yorkshire and The Humber–South Yorkshire Research Ethics Committee (IRAS: 230098). Participants provided written consent to take part in the study.

### Participants

18 participants were recruited using opportunity and snowball sampling (3 men, 15 women) aged 20 to 39 (M: 28.44; SD: 5.52) in 2021. The former was identified as an appropriate sample size to reach sufficient data saturation and serve the requirements of the objectives and research design utilized in this study [19]. UK universities were asked to circulate email invitations to their students and staff, and posts were advertised on social media platforms including Twitter, Facebook and Instagram.

Participants were aged 18 or older, of South Asian ethnicity (nine were of Pakistani ethnicity, six Indian, two Bangladeshi and one Afghan), and residing in the UK. Nine participants were born outside of the UK but had spent 13.4 years on average living in the UK (range = 2–31 years) They needed to have a significant other (i.e. child, partner, parent, sibling, etc.) with whom they had regular contact and who they could answer the questions in relation to. Ten participants answered in relation to their parent, five about their partner (husband, fiancé or boyfriend), two about a sibling or sister-in-law and one about their offspring. If participants or their significant others had any significant mental or physical health problems (i.e. Received treatment or diagnosis from a practitioner), they were excluded from the study with the aim of obtaining normative data in the absence of illness-related stressors.

Participants were compensated with £10 Amazon vouchers for their participation.

### Measures

A semi-structured interview was conducted by master’s and doctoral level researchers. The interview schedule consisted of five open-ended questions exploring EE in participants’ relationships with their ‘significant others’. The schedule followed the ethos of commonly used tools to identify EE i.e. the Five-Minute Speech Sample [20] and Camberwell Family Interview [21] which use an exploratory approach to understand the nature of both positive and negative aspects of relationships. Existing EE domain terms (eg. Emotional over-involvement, criticism) were not included in the questions to allow for an inductive exploration of themes. Participants were requested to think of a person they have a close relationship with when answering questions. This person could be a.) someone that you live with or have done in the recent past b.) a partner or relative (e.g., boyfriend/ girlfriend/ spouse/ child/ parent/ sibling/ niece/ nephew/ uncle/ aunt/ grandparent/ grandchild etc.) c.) someone who if they were to become physically or mentally unwell, you would take a significant role in caring for them.

Interview questions consisted of: What’s your relationship with X like; What aspects do you like about your relationship with X; What aspects do you not like about your relationship with X; Have you noticed any changes in your relationship over time; Is there anything in your relationship with X that you would like to change/be different. Interviewers were trained to prompt participants with open-ended verbal cues.

The interview was piloted with three individuals to determine the order of the questions, whether they were understandable and fit for purpose, and if the interview completion time was appropriate. Based on feedback, positive aspects of the relationship were explored first, and additional probes were added to elicit more information where necessary. Questions that were originally included, exploring whether culture impacts on the relationship, were removed in the interest of keeping completion time low. It was felt these questions might be leading or biasing participants.

#### Procedure

Interviews lasted 26 minutes on average (*Min* = 10min 16s; *Max* = 44min 12s), were conducted online via Microsoft Teams, and audio/video recorded. Reflexivity journals were maintained by the interviewers throughout the study process. They also partook in a bracketing interview to engage with the assumptions they were bringing to the process. This was intended to create transparency regarding their position in relation to the research and to remain aware of any potential influences that they may have on the data.

### Data analysis

Interview transcriptions were attained through Microsoft Teams and proof-read for errors. They were entered into QSR NVivo 7 and analysed using principles of Thematic Analysis [22]. This involved six stages: familiarisation, initial coding, searching for themes, reviewing themes, defining, and naming themes and producing the report. Data analysis was conducted using an inductive, bottom-up approach; themes were identified based entirely upon the data collected rather than on pre-existing theoretical concepts. This is highlighted by the first iteration of themes (Table 1) where theme names were generated based on actual quotations from the data.

**Table 1:** Table of themes before and after iteration process.

First iteration	Final iteration
1. There is value in setting boundaries 2. Actions speak louder than words 3. “She’s really adapted to the culture 4. “I would keep her health over my own” 5. “Like my best friend” 6. “She can be mean and harsh and super critical” 7. “That’s what makes her her”	1. Expression of love 2. Setting boundaries 3. Inter-generational differences 4. Acceptance

Generated themes were individually reviewed by the authors in two group discussions before being finalised. This process involved merging overlapping themes and devising more succinct theme names.

## Results

**Fig 1:**
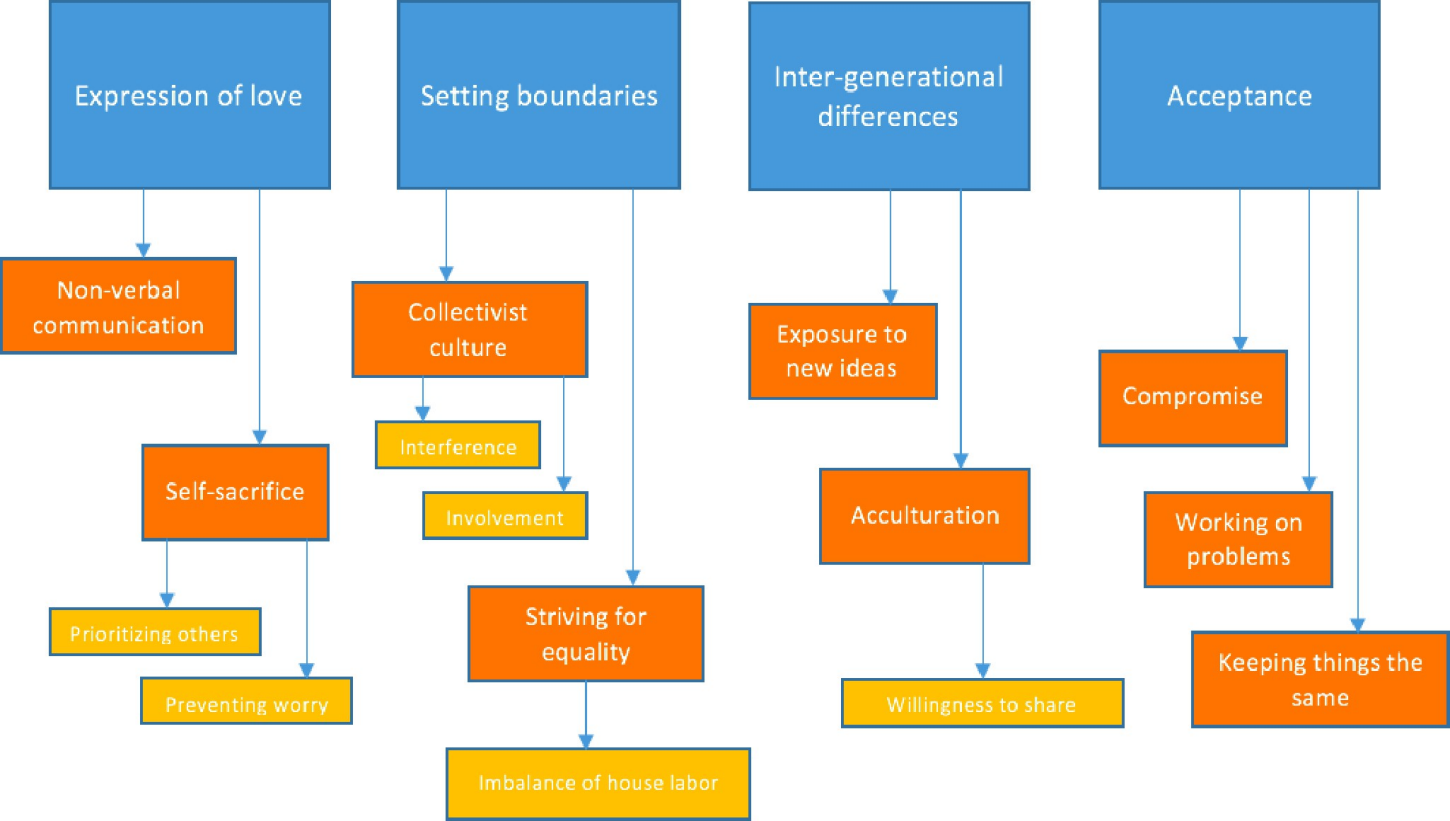
Thematic map. Main themes: Blue; Intermediary themes: Orange; Subthemes: Yellow.

The data generated four main themes: expression of love, setting boundaries, inter-generational difference, and acceptance. Themes were further categorised into intermediary and sub-themes (Fig 1). Quotations exemplifying each theme are presented in the corresponding tables.

### 1. Expression of love (Table 2)

**Table 2:** Quotations illustrating “Expression of love”.

Main Theme	Sub-theme	Quotation
**Non-verbal communication**		And just the fact that she really loves me because, you know, sometimes parents don’t always express that, but even though she may not say it specifically, I know that she loves me in the way that she is constantly looking out for me and checking in with me and, just even asking what did I eat today? What did I cook today? It just lets me know that she’s thinking of me which is really nice. [P16: 29 y/o; woman; Muslim; Bangladeshi; speaking about relationship with mother]
**Self-sacrifice**	Prioritising others	The word selfish is used a lot because it’s her way of saying “you’re doing things for yourself. You shouldn’t. You should just do things for the whole family”. [P11: 27 y/o; woman; Muslim; Bangladeshi; speaking about relationship with sister]
	Preventing worry	I try not to worry her with my worries or with any other things and so even when she’s asking “how are you?” I just say everything is good and it’s no problem so I try not to share too much and sometimes when I’m holding back, that’s difficult as well ’cos, you know, as a daughter, you’ve always shared your smallest to biggest problem problems with your mother. And I mean we’ve always been very close, and we shared everything with her so it’s difficult to hold back and not tell her that this is the problem I’m having these days. [P15: 36 y/o; woman; Muslim; Pakistani; speaking about relationship with mother]

#### Non-verbal communication

Participants described a lack of overt expression of love within their families. Whilst they were certain ’love’ was present, they explained how it was rare for it to be communicated explicitly. Instead, love was expressed in non-verbal ways such as constantly looking out for and checking in with your significant others and through spending time and engaging in activities with each other. Participants responded to this lack of verbal expression in one of two ways. Some felt they were lacking something and hoped for it to change. For others, they were content with the unspoken display of affection and felt that the implicit expression of care offered enough security in their relationship, and they were able to experience love and support.

#### Self-sacrifice

Participants described making personal sacrifices for their significant others, owing to a sense of duty which they felt towards their family; this was not specific to any relationship. Whilst acts of self-sacrifice are viewed unfavourably due to their association with poorer mental health outcomes [23], these were a common way of expressing love in South Asian relationships. When individuals did not perform self-sacrificial acts, this was looked down upon.

### 2. Setting boundaries (refer to Table 3)

**Table 3:** Quotations illustrating “Setting boundaries”.

Main Theme	Sub-theme	Quotation
**Collectivist culture**	Interference	They get offended when I put the boundary forward and say you’re crossing a line over here. If we want a civil relationship, you have to step back a little bit, but they find that very offensive. I’m from, like I said, a Pakistani background so boundaries do not exist in Pakistani families is what I’ve found… so when you do put boundaries down, it’s seen as being offensive. I guess it maybe just comes from the fact that Pakistan is a collective culture, whereas a lot of us in the UK, we come from more of an individualistic culture so maybe it’s that clash. [P06: 25 y/o; woman; Muslim; Pakistani; speaking about relationship with mother-in-law]
	Involvement	I think when she advises me, she advises me in a manner that’s not very intrusive, so it kind of makes me think rather than “this is the way you should be living your life” and so I really appreciate that approach from her, especially now as a married woman. She’s been married for over 30 years, so she could very easily say “I know more than you and you’ve been married only a couple of years” but actually the way that she communicates with me is really nice [P16: speaking about relationship with mother]
**Striving for equality**		Like I mentioned, it used to be a lot more authoritarian where she says what she says and we have to do it. And if we don’t do it, it’s game over. But now it’s more like she’s weakened the grip basically and she’s kind of said “go live in the world” and I can kind of see that she literally lets me do whatever I want and that was an outlandish thought maybe 2–3 years ago… When we were younger it was just “no, whatever I say goes” and now she’s dealing with the fact that we’re going to do things and she’s not going to agree with them but we could still do them, you know. So the flexibility has definitely been there that I didn’t expect.
		[P14: 21 y/o; man; Muslim; Pakistani; speaking about relationship with mother]
	Imbalance of house labour	Why I’m not liking that that’s the situation? Obviously because I do the majority of it which I think is unfair because if I had not been a student at university, I’ve been working full time also and obviously he’s been working full time so to me it feels unfair that I’m working full time hours and I’m coming home and doing everything by myself so it kind of feels like doing a double shift and I’m constantly working in my life other than when I’m asleep. So it feels like too much. It’s really unfair. So yeah. It’s never something that I’ve been shy talking about either. This is something that he’s known from the beginning of our relationship [P06: 25 y/o; woman; Muslim; Pakistani; speaking about relationship with husband]

#### Collectivist culture

Participants described how coming from a collectivist culture meant that families were accustomed to being involved in each other’s lives. Whilst they considered a certain level of involvement acceptable, they did not appreciate *interference* into their daily lives and relationships, possibly highlighting the role of cultural assimilation/acculturation and generational differences.

Problems in relationships primarily emerged when family members were getting overly-involved in their personal lives. This included when individuals did their relatives’ everyday tasks for them, emotionally manipulated them to act in a certain way or offered their opinions on relationships they were not a part of. For instance, *P06* described how her mother-in-law would get involved in her relationship with her husband and took offence when she set boundaries to prevent this. Similarly, *P10* (39 y/o; woman; Pakistani; Muslim) explained how her mother would direct her daughter to act in ways which did not align with her own values. These examples a level of interference or control in these relationships.

Individuals valued *involvement* from their significant others in a friend-like way; positive relationships were “*honest*” and “*informal*”, and those where they could “*banter*” with each other. They appreciated advice from their significant others in a non-intrusive, lenient manner which still granted them autonomy in decision making.

#### Striving for equality

Perhaps due to the collectivist culture, parents stayed involved in their offspring’s lives for longer than they ordinarily would in individualist cultures. Participants described negotiating boundaries with their parents as they got older to make their relationship more adult-to-adult, highlighting the gradual transition from authoritarian parenting to a more equal relationship. The ability to agree-to-disagree provided a good foundation to reduce control and increase flexibility in the relationship.

Participants’ appreciation for the role their parents’ played when they were younger increased with age. As teenagers, they described feeling like their parents were being intrusive or over-protective. As adults, they felt that they could better understand why their parents were like this, suggesting this cohort does value their relatives being involved in their lives.

Women in romantic relationships commonly spoke about the imbalance of house labour as a cause of disagreement, indicative of gender inequality. Especially when both partners were in full-time employment, they felt it was unfair for the household responsibilities to fall on the woman partner. They described being open with their partners about their disapproval for this inequality, another way in which boundaries were set.

### 3. Inter-generational differences (refer to Table 4)

**Table 4:** Quotations illustrating “Inter-generational differences”.

Main Theme	Sub-theme	Quotation
**Exposure to new ideas**		I feel like he’s a very rigid person still. Again, I think this is going to the whole … it’s a very South Asian thing, but like in terms of his views of the world, I feel like it’s very fixed in place and. It’s not necessarily always open to different sorts of ideas. I think that’s more of a change in our generation that we’re a lot more (open) obviously, ’cos we’re exposed a lot more to different values and different ways of seeing things which their generation might not necessarily have been, so that’s maybe a slight difference between us and them. [P18: 24 y/o; man; Pakistani; Muslim; speaking about relationship with father]
**Acculturation**		The person that she was when she first came to this country is different to the person she is now, and it’s all those experiences of being a young adult in this country, being a married woman, raising six children that have gone through their education process, university, work, getting married here. All of those experiences in the UK have adapted her mind-set and that’s also made it easier for me to talk to her because you know, things like boys and friendship issues or money problems or you know things that you know you might find that occur in the UK now, my mom’s a lot more understanding of what goes on here. She’s more clued up if that makes sense. [P16: speaking about relationship with mother]
	Willingness to share	She’s really adapted to the culture and the ways that people are here. Just really more open minded and accepting of how things are at the moment, so I think that that’s kind of helped her have be a bit easier to talk to when it comes to things like that… I guess if her mind-set was a bit more… I don’t want to say backwards, but like I guess if it wasn’t so current. ‘Cos I feel like some people from Pakistan are very cultured and they’re set in their ways. So the fact that she’s not like that, I think that’s helped because it just means that she’s able to understand more what it’s like more as being as being a kid kind of growing up here, as opposed to just thinking that I should be growing up the way that she grew up [P05; 26 y/o; woman; Pakistani; Muslim; speaking about relationship with mother]

#### Exposure to new ideas

Participants explained how elder family members were often quite stubborn in their thinking; they struggled to accept their mistakes or understand other/alternative perspectives. Participants who had grown up in the UK attributed this rigidity to a generational gap. Suggestive of acculturation processes, they felt that they were more open to accepting new ideas as compared to their parents because of the increased exposure they had to other ways of living.

The importance of respect for elders in a South Asian context was also recognised. Regardless of who was factually correct, younger individuals described how they would sometimes have to dismiss their own viewpoints as a sign of respect for their older relatives; these ideas link back to themes of self-sacrifice.

#### Acculturation

A key factor which affected the relationship participants had with their parents was the extent to which they had adapted to Western culture. Having experienced life in the UK, participants described how their parents had become more “*modern*” and “*liberal*” *(P07*: *37 y/o; woman; Pakistani; Muslim; speaking about relationship with father)*, differing from the general conservative stance most South Asian parents held. It is likely that having lived in both South Asia and the UK, these parents held views aligned with value systems which incorporated both traditional more collectivist and ‘western/individualistic’ concepts. This therefore reduced the extent to which their viewpoints differed from their offspring, and allowed them to be more open about their thoughts and experiences.

Whilst openness was a characteristic which many individuals described as crucial for a healthy relationship, offspring were still mindful about what they shared with their parents. This was especially the case when thoughts or behaviours did not align with their parents’ cultural expectations. There was seemingly more openness in parent-offspring relationships where parents were more willing to tolerate differences in viewpoints and agree-to-disagree.

### 4. Acceptance (refer to Table 5)

**Table 5:** Quotations illustrating “Acceptance”.

Main Theme	Quotation
**Compromise**	I would say, like initially, or at least when we first started, kind of living together, it was a little more difficult because it was quite frustrating having to have expectations that weren’t being met. But I think overtime we’ve both changed in the sense that I have started to expect different things and I don’t have as high expectations or as strict expectations, and he’s also kind of adapted to what I prefer. And yeah, just some natural changes happened overtime. [P09: 20 y/o; woman; Indian; Hindu, not practicing; speaking about relationship with boyfriend]
**Working on problems**	We don’t hold grudges against each other so if one of us has done something to upset the other, we will just tell them straight away there’s no sort of playing around guess what I’m thinking. In our relationship and sometimes that does mean that we’re talking for hours and hours and hours trying to understand the other person’s viewpoint. But I would rather do that then not know what not knowing what the other person is doing and just having resentment build up. [P06; speaking about relationship with husband]
**Keeping things the same**	I think like for me I always kind of understand that she has grown up differently and that her culture is part of her and I get that she like, although she’s moved here and she’s living here and she has adapted really well, it’s still going to be instilled, like certain things in the way that she might have gone about doing things in Pakistan are still kind of there. So I feel like although it’d be easier for me, maybe if things could change I think I still recognise the fact that that’s part of her, so I wouldn’t change it really ’cos it’s what makes her her. [P05: 26 y/o; woman; Pakistani; Muslim; speaking about relationship with mother]

#### Compromise

Participants discussing romantic relationships commonly recognised that they would have to accept certain details about their partner and their lifestyles to make the relationship work and vice versa. They became more understanding of their partners’ views and actions over time; this helped strengthen their relationship.

#### Working on problems

When problems arose in relationships, participants described how they tried to acknowledge and address them immediately. This was the case in all relationships, romantic and otherwise. Participants valued being able to converse with their significant other about their feelings and needs. They appreciated that their significant others were open to these conversations, and that they could work on making these changes together. Interestingly, this is not the case cross-culturally; other communities value space for reflection before they address a problem [24].

#### Keeping things the same

Interestingly, participants explained how although they disliked certain aspects about their significant other, for instance rigidity or conservative thinking, they did not want these things to change. They worried that changing these traits would affect their relationship.

## Discussion

This study provides an understanding of EE in UK-based South Asian family relationships. It uses an inductive approach to examine how emotions are expressed within this population. These findings provide an awareness of South Asian cultural norms in the UK, in relation to caregiving and receiving behaviours. They contribute to the wider literature on understanding EE through a cultural lens.

Corroborating Hashemi and Cochrane’s [18] report, this study showed that acts of self-sacrifice were common in South Asian families even in the absence of significant health stressors. This raises the question of whether self-sacrificial behaviours in this population are pathological or merely an acceptable way of expressing love. The latter explanation is supported by research suggesting that Pakistani children are raised in a way that encourages closeness and sacrificial behaviours for their relatives [25]. In fact, the absence of personal sacrifices and other acts of EOI was perceived as a lack of care [26]. Therefore, self-sacrificial acts in South Asian communities may not actually warrant the same level of concern as they would in other socio-cultural contexts where they may be indicative of subjugation and denying one’s own needs to please others [23].

The intermediary theme ‘acculturation’ highlighted how participants’ relationship with their parents was affected by differences in their bicultural identities. When these were similar, participants were more open. Previous literature established an association between generational status (first vs second vs later) and varying acculturation preferences. It found that second-generation Pakistani immigrants favoured an integration strategy compared to their first-generation counterparts who preferred separating from the dominant culture [27]. Evidently, these differences impact the expectations individuals have of their relationships. Those who held on to their cultural values had corresponding expectations, such as greater involvement or self-sacrificial behaviours from their significant others. Conversely, those who acculturated more had expectations resembling local values of privacy, autonomy and individualism. This incongruity impacted relationships as individuals had trouble meeting the other’s expectations. Nevertheless, this study found that South Asians held on to certain values (for example, respect for elders) which allowed them to accept, and even appreciate, these differences. The themes of compromise and negotiating and accepting differences are very prevalent in the South Asian diaspora in the UK, especially among the second generation. They reflect the struggles of navigating a bicultural identity and intergenerational differences; these concepts are important to consider when delivering clinical interventions such as family interventions, and to aid open communication between family members regarding these disparities with the use of psychoeducation and normalization

South Asian families displayed many of the behaviours Brown et al. [28, 29] identified as indicators of warmth. They displayed concern by looking out for and checking up on each other and expressed interest through spending time with their significant others. Only one participant talked about the use of explicit positive remarks in her family. The majority discussed how love was not verbalised in their households, but instead shown in non-verbal ways including involvement, not interference, and self-sacrifice. This corroborates previous research which showed that Pakistani families expressed fewer positive remarks on average compared to those in other communities and highlights cultural variations in the expression of warmth [30].

In collectivist cultures, an absence of positive remarks could have more negative repercussions than the presence of criticism, which might instead be indicative of care and support. Thus tailoring clinical interventions to increase the expression of positive remarks and enhance positive feelings and resilience instead of attempting to reduce criticism might be helpful in this sociocultural context [31].

Participants valued and even expected a degree of involvement from their relatives but distinguished welcomed involvement from interference. They wanted their significant others to take interest in their lives, give them advice and be a source of encouragement. This can be attributed to their collectivist culture [32] where closeness and interdependence are expected in relationships. These communities place family at their core and value their individual needs as secondary to their family duties [33]. The distinction between intrusive and non-intrusive involvement is of importance as the former is predictive of psychosis relapse [14]. This finding was corroborated by participants in this study who appreciated involvement but not interference.

Only two participants described experiencing criticism and hostility from their significant others. *P11* described “*harsh*, *mean comments*” from her sister which stemmed from her irritation at her doing more for herself than for the whole family. Similarly, *P03* described passive aggressive comments from her mother about the limited time she spared for her. Both examples demonstrate relationship difficulties stemming from participants placing their own needs before their families. This links to intergenerational differences, highlighting a move away from the expectations of being self-sacrificing and dutiful towards your family to having a more individualistic stance. It also highlights how in the absence of neutral or positive aspects of EE, criticism and hostility can have negative consequences on relationships; a finding which appears to apply cross-culturally [33].

### Limitations

These findings may have been biased by gynocentric views and perceptions. Research has suggested that women display higher emotional over-involvement than men [9] because of the caretaking and attachment role traditionally attributed to them. Although the views of the men corresponded with the women, different themes may have emerged in a less gender-biased sample.

The lack of representation across South Asian cultures in the sample could be another limitation that did not allow exploration of within-group variation. Since participants from Nepal, Sri Lanka, Bhutan or the Maldives could not be recruited, it cannot be determined whether their views were represented in our findings. Despite the similarities between South Asian cultures, people in these regions may still differ owing to factors like religion or politics. Even in terms of EE, for instance, studies have shown that Pakistani families had higher EE than Indian families [18].

#### Implications and future research

These preliminary findings help to distinguish between behaviours that are pathological and customary in UK based South Asian families. For example, characteristics such as ‘involvement’ and ‘self-sacrifice’ in South Asian caregiving relationships may serve protective functions. Equally, findings highlight the impact of navigating and negotiating bicultural identities, generational differences and degrees of acculturation in EE in the South Asian context. As a result, it may be useful to envisage EE on a spectrum from traditional and collectivist cultures to the more modern and individualist cultures. This would suggest that the boundaries between cultures are perhaps not clear-cut, especially in migrant groups and across generations.

Future research should consider a more in-depth investigation and explore the ecological validity of EE across cultures in real-world settings. These findings can be applied to explore caregiving relationships in South Asian families that are facing physical or mental health stressors and investigate healthcare professionals’ perspectives and experiences of working with UK-based South Asian families and potentially facilitate the development of culturally specific adaptations to clinical interventions.
